# Normal pressure hydrocephalus and CSF tap test response: the gait phenotype matters

**DOI:** 10.1007/s00702-020-02270-3

**Published:** 2020-10-26

**Authors:** Eric Morel, Stéphane Armand, Frédéric Assal, Gilles Allali

**Affiliations:** 1 Department of Neurology, University Clinic of Neurology, Inselspital, Bern University Hospital, University of Bern, Freiburgstrasse 16, 3010 Bern, Switzerland; 2 Faculty of Medicine, University Geneva Hospitals, University of Geneva, Rue Gabrielle-Perret-Gentil 4, 1205 Geneva, Switzerland; 3 Willy Taillard Laboratory of Kinesiology, University Geneva Hospitals, Geneva, Switzerland; 4 Division of Neurology, Department of Clinical Neurosciences, University Geneva Hospitals, Geneva, Switzerland; 5 Department of Neurology, Albert Einstein College of Medicine, Yeshiva University, Bronx, New York, USA

**Keywords:** Gait disorders, Normal pressure hydrocephalus, Clinical evaluation, Gait phenotypes

## Abstract

This study compared gait speed changes after CSF tap test in patients with idiopathic normal pressure hydrocephalus presenting with various gait phenotypes (frontal, parkinsonian, normal, or other). All patients improved, except those with parkinsonian gait.

## Introduction

Gait disorders are the hallmark feature of patients with idiopathic normal pressure hydrocephalus (iNPH) (Relkin et al. 2005). INPH patients present various gait phenotypes from normal gait to severe frontal or parkinsonian gait (Morel et al. 2019). The variability of these phenotypes may be due to the severity of iNPH or influenced by comorbid neurological (i.e., vascular lesions) and non-neurological (i.e., arthritis) diseases (Stolze et al. 2001). To assess the reversibility of gait impairment in iNPH, the CSF tap test represents a widely used prognostic procedure (Krauss and Halve 2004). However, the influence of gait phenotypes on gait changes after CSF tap test has never been studied in patients with iNPH. Therefore, we aimed to compare gait speed changes after CSF tap test among the various gait phenotypes presented by patients with iNPH. Establishing which gait phenotype in iNPH is associated with good clinical outcomes after CSF tap test may improve the identification of appropriate candidates for shunt surgery.

## Methods

A total of 77 consecutive iNPH patients from the Geneva iNPH cohort (Allali et al. 2017) were included in this retrospective study (age 76.1 ± 6.2 years; 32.5% female). Study procedures have been previously described (Allali et al. 2017). Briefly, patients were referred for suspicion of iNPH based on gait disturbances, cognitive impairment, and/or urine incontinence. Inclusion criteria were patients with a diagnosis of possible or probable iNPH (i), able to walk without assistance (ii), a video recording of their gait pre-CSF tap test (iii), and a measure of gait speed pre- and post-CSF tap test (iv). Exclusion criteria were any acute medical condition in the 3 months before the examination and a diagnosis of secondary NPH. INPH was diagnosed according to the international criteria (Relkin et al. 2005) after a case conference involving neurologists, neuropsychologists, and physical therapists (possible iNPH in 87%; probable iNPH in 13%). Gait phenotypes were evaluated by two assessors (EM and GA)—blind for the gait changes after CSF tap test—with a substantial agreement (kappa, 0.73), who classified the phenotypes as normal, frontal, parkinsonian, and other. As previously described (Verghese et al. 2002), frontal gait was defined by short steps, a wide base of support, and a magnetic component (reduced step height); parkinsonian gait was defined by short and/or shuffling steps, flexed posture, reduced arm swing, and normal base; normal gait was defined by the absence of any clinical gait abnormalities, and other gait was defined by any other clinical gait abnormalities (Morel et al. 2019). Patients walked at their comfortable speed on a 10-m walkway before the CSF tap test and 24 h after, as previously suggested (Virhammar et al. 2012). Walking speed was calculated from the trajectory of reflective markers attached to the patient's heels, evaluated by an optoelectronic system (Vicon Mx3+, Oxford Metrics, UK). Statistical analysis used ANOVA or Kruskal–Wallis test, as appropriate, to evaluate differences between gait phenotypes. Gait speed changes were calculated according to the following formula: (gait speed_post-CSF tap test_ − gait speed_pre-CSF tap test_)/[(gait speed_post-CSF tap test_ + gait speed_pre-CSF tap test_)/2]. Univariable linear regressions evaluated the relationship between gait speed changes (dependent value) and each gait phenotype (independent value). Multivariable linear regressions were adjusted for age, gender, comorbidities, white matter changes assessed by the age-related white matter change scale (Wahlund et al. 2001), and mini-mental state examination (MMSE). The Ethical Review Board of the Geneva University Hospitals approved the study (Protocol 09-160R).

## Results

**Fig. 1:**
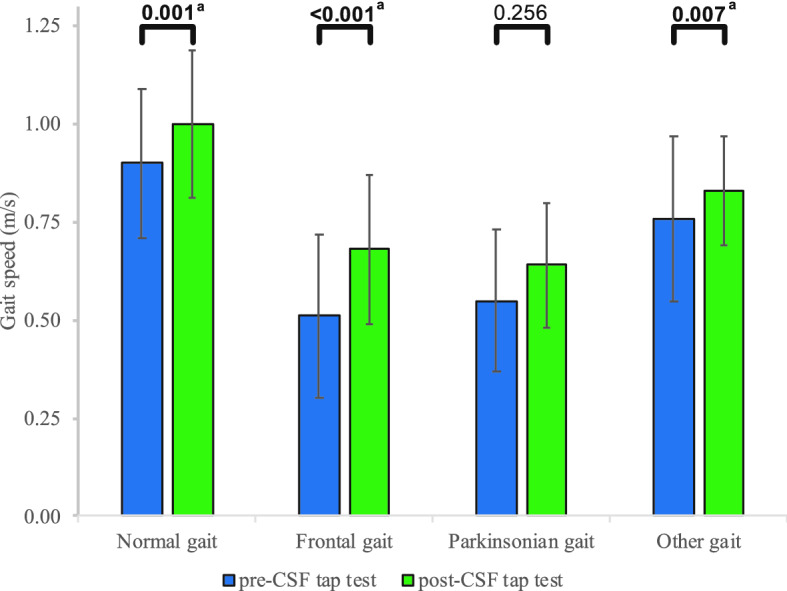
Gait speed changes. Gait speed changes are presented before and 24 h after CSF tap test. ^a^Significance level was set at *p* < 0.05

Clinical characteristics are presented in Table 1: 30% of the patients presented with a normal gait, 25% with a frontal gait, 15% with a parkinsonian gait, and 30% with other gait abnormalities. Global cognition and white matter changes were similar across the various gait phenotypes. The average gait speed of the cohort was relatively slow (0.71 ± 0.25 m/s); patients with frontal and parkinsonian gait were the slowest walkers (0.51 ± 0.18 m/s and 0.55 ± 0.18 m/s, respectively). Except for those with parkinsonian gait, all patients significantly improved their gait speed after CSF tap test (Fig. 1). Patients with frontal gait improved their gait speed to a greater extent in comparison to other gait phenotypes (delta: 0.31 ± 0.31; *p* value < 0.001). Frontal gait was significantly associated with gait speed improvement after CSF tap test, even after adjusting on age, gender, comorbidities, white matter changes, and MMSE (*β*: 0.311 [95% CI 0.058; 0.334]; *p* value: 0.006). Normal gait, parkinsonian gait, and other gait abnormalities were not significantly associated with gait speed improvement, after adjusting on age, gender, comorbidities, white matter changes, and MMSE (normal gait: *β*: − 0.197 [95% CI − 0.262; 0.028]; *p* value: 0.112; Parkinsonian gait: *β*: 0.031 [95% CI − 0.150; 0.197]; *p* value: 0.788; other gait abnormalities: *β*: − 0.160 [95% CI − 0.241; 0.046]; *p* value: 0.179).

**Table 1:** Characteristics of the participants (*n* = 77)

	Normal gait (*n* = 23)	Frontal gait (*n* = 19)	Parkinsonian gait (*n* = 12)	Other gait (*n* = 23)	*p* value
Age, years	75.13 ± 7.88	76.89 ± 4.69	75.08 ± 5.74	76.00 ± 5.76	0.717
Gender, *n* (% female)	7 (30.4)	5 (26.3)	3 (25.0)	10 (43.5)	0.590
Disease duration, months	29.09 ± 23.77	43.79 ± 38.67	40.50 ± 20.33	42.75 ± 31.54	0.319
Comorbidity (0–10)^b^	1.26 ± 1.01^a^	2.00 ± 1.16	2.00 ± 1.04	2.25 ± 0.79	0.008*
Medication, *n*	2.87 ± 2.30^a^	4.42 ± 1.17	4.83 ± 2.73	4.15 ± 2.46	0.047*
Mini-mental state (0–30)	26.30 ± 4.99	25.68 ± 4.88	24.67 ± 5.33	26.78 ± 3.63	0.704
Risk factors					
Vascular (0–5)^c^	1.13± 0.87	1.26 ± 0.73	1.50 ± 0.80	1.50 ± 1.15	0.675
Cardiovascular (0–4)^d^	0.04 ± 0.21^a^	0.37 ± 0.50	0.08 ± 0.29^a^	0.25 ± 0.44	0.028*
Cerebrovascular (0–2)^e^	0.04 ± 0.21	0.16 ± 0.38	0.17 ± 0.39	0.00 ± 0.00	0.154
White matter changes^f^					
Frontal (0–6)	2.65 ± 1.80	2.21 ± 1.51	2.08 ± 1.38	2.45 ± 1.40	0.741
Parieto-occipital (0–6)	2.26 ± 2.12	1.89 ± 1.85	1.83 ± 1.64	2.40 ± 1.67	0.677
Temporal (0–6)	0.61 ± 1.27	0.42 ± 1.07	0.75 ± 1.42	0.80 ± 1.32	0.702
Basal ganglia (0–6)	0.52 ± 0.85	0.58 ± 0.96	0.58 ± 0.79	0.45 ± 0.83	0.969
Infratentorial (0–6)	0.26 ± 0.62	0.26 ± 0.65	0.00 ± 0.00	0.30 ± 0.66	0.480
Total score (0–30)	6.30 ± 5.45	5.37 ± 4.21	5.25 ± 3.84	6.40 ± 4.80	0.814
Gait speed					
Pre-CSF tap test (m/s)	0.90 ± 0.19^a^	0.51 ± 0.21	0.55 ± 0.18	0.76 ± 0.21	< 0.001*
Delta (m/s)^g^	0.12 ± 0.14	0.31 ± 0.31	0.17 ± 0.47	0.10 ± 0.14	0.053

Results are given in mean ± standard deviation, unless indicated otherwise

*Significance level was set at *p* < 0.05

^a^Patients presented significant differences in comparison with the other groups

^b^Diabetes, chronic heart failure, hypertension, depression, stroke, Parkinson disease, chronic obstructive pulmonary disease, angina, and myocardial infarction

^c^Diabetes, hypertension, hypercholesterolemia, body mass index > 30, and smoking

^d^Myocardial infarction, angina, arrhythmia, and chronic heart failure

^e^Transient ischemic attack and stroke

^f^Age-related white matter changes quantify the burden of white matter disease in the whole brain and its subregions

^g^Delta of gait speed was calculated as follows: [(gait speed post-tap test) − (gait speed pre-tap test)]/[(gait speed pre-tap test) + (gait speed post-tap test)]/2)

## Discussion

This study showed that gait improvement after CSF tap test varies across gait phenotypes: frontal gait is associated with the greatest gait improvement after CSF tap test, while patients with parkinsonian gait did not show any significant gait speed changes.

In comparison with other gait phenotypes, iNPH patients with frontal gait dramatically improved their gait speed after CSF tap test (delta: 0.31 ± 0.31; *p* value < 0.001). Patients with frontal gait presented the lowest gait speed at baseline and, therefore, had the greatest potential of improvement, as previously described (Kahlon et al. 2007). The present findings partially contrast with a previous study, showing that hypokinesia responds more to CSF tap test than frontal signs including disequilibrium (Bugalho and Guimarães 2007); in comparison with this previous study, we focused here on gait phenotypes and not on individuals neurological signs. Furthermore, the patients included in Bugalho’s study were more severely affected (mean gait speed: 0.33 ± 0.20 m/s) in comparison to our study (0.71 ± 0.25 m/s). The role of irreversible comorbid neurological conditions may also explain these differences in terms of gait reversibility after CSF tap test: the presence of parkinsonism in patients with higher level gait disorders (such as iNPH) has been associated with Alzheimer’s pathology (Allali et al. 2018). Another explanation for this discrepancy in CSF response between iNPH patients with frontal and parkinsonian gaits may refer to the brain structures and the pathogenesis associated with both these gait phenotypes: older adults with parkinsonian gait present more severe executive deficits in comparison to those with frontal gait, suggesting the involvement of different brain regions between both these gait phenotypes (Ambrose et al. 2006).

Patients with a clinical phenotype of normal gait also demonstrated a gait improvement after CSF tap test. The relatively high proportion of patients with normal gait (30%) may be explained by the following reasons. First, patients were included at an early stage of the disease course, where clinical gait abnormalities may not be evident. Second, patients have been referred, because the suspicion of iNPH was solely based on cognitive impairment and/or urinary incontinence. Third, patients may complain of gait impairments in challenging situations (e.g., uneven ground) or balance impairments that were not clinically evident in the secure setting of a gait laboratory. Fourth, they presented fewer comorbidities that may affect gait. These results suggest that CSF tap test could also be considered at the earliest stages of iNPH when patients complain about their gait, but no clinical gait abnormality is diagnosed by physicians.

The variability of the gait phenotypes in our cohort of iNPH patients may be explained by comorbidities (Stolze et al. 2001). Neurological (i.e., Alzheimer’s disease or cerebrovascular disease) and non-neurological (i.e., osteoarthritis) comorbidities likely contribute to each pathological gait phenotype, as highlighted by the highest score of comorbidities in the pathological gait phenotypes.

The main strength of this study is confirming the interest and the validity of a non-expensive clinical approach (without any gait analysis system but only the clinician’s eyes). However, the validity of the clinical examination (i.e., clinical gait characteristics) is not perfect and prone to an interrater variability. Having a better quantification of neurological and non-neurological comorbidities would allow a better sense of the influence of comorbidity on each gait phenotype. A post-mortem pathological examination is missing and would be of interest, especially in this cohort including mainly patients with possible iNPH (87%), who may present either a comorbid neurological condition along with iNPH or present an iNPH mimic. Finally, future studies should confirm these results by evaluating the predictive value of clinical gait phenotypes after shunting.

## Conclusion

Among gait phenotypes, frontal gait in patients with iNPH is associated with the largest gait improvement after CSF tap test. This study suggests that a clinical classification of gait phenotypes in patients with iNPH may inform about the reversibility of gait disabilities. Future studies should include long-term clinical outcomes after shunt procedure to confirm that frontal gait in iNPH patients may present a good clinical outcome in comparison to other gait phenotypes.

## Data Availability

The data are kept by the first author.
